# The prevalence of insomnia in different COVID-19 policy phases: Longitudinal evidence from ITA.LI – Italian Lives

**DOI:** 10.1186/s12889-022-14048-1

**Published:** 2022-09-01

**Authors:** Egidio Riva, Marco Terraneo, Mario Lucchini, Tiziano Gerosa

**Affiliations:** 1 Dipartimento Di Sociologia E Ricerca Sociale, Università degli Studi di Milano – Bicocca, Via Bicocca degli Arcimboldi, 8, 20126 Milano, Italy; 2 University of Applied Sciences and Arts of Southern Switzerland, Institute of Applied Sustainability to the Built Environment, Via Flora Ruchat-Roncati, 15, CH-6850 Mendrisio, Switzerland

**Keywords:** Sleep disturbance, Trajectories, Panel study, Italy, Pandemic, Containment policies

## Abstract

**Background:**

This study investigated changes in the prevalence of insomnia in Italy during COVID-19, starting from the first lockdown period (8 March 2020). We hypothesized that lockdown precipitated increased prevalence of insomnia symptoms relative to the pre-pandemic period; b) the gradual relaxation of containment measures – post-lockdown period (Phase 2 and Phase 3) – reduced insomnia severity, leading to a relative recovery of pre-pandemic levels; and c) we tested age-related heterogeneity in sleep responses, with an expected higher increase in insomnia in younger and middle-age groups.

**Methods:**

Analyses drew on a subsample (*N* = 883) of respondents to ITA.LI – Italian Lives, a recently established panel study on a probability sample of individuals aged 16 + living in Italy. To estimate patterns of change in insomnia, we first fitted a random-effects ordered logistic model on the whole sample. We then added an interaction term between policy phases and the respondent age to test whether the relationship between insomnia and policy phases differed across age groups. Analyses accounted for survey non-response weights.

**Results:**

The fraction of respondents reporting moderate (“somewhat” + 0.159, S.E. 0.017) or severe (“very much” + 0.142, S.E. 0.030) sleep disturbances significantly increased during Phase 1. The prevalence of insomnia followed an inverted U-shaped curve across policy phases, with further increases from baseline levels (“somewhat” + 0.168, S.E. 0.015; “very much” + 0.187, S.E. 0.030) during Phase 2, followed by a relative reduction in Phase 3, although it remained significantly higher than in the pre-pandemic period (“somewhat”, + 0.084, S.E. 0.016; “very much”, + 0.045, S.E. 0.010). There were significant age-related differences in insomnia patterns, as the discrete change from pre-pandemic levels in the probability of not suffering from insomnia was negative and significant for the younger age group (− 0.269, S.E. 0.060) and for respondents aged 35–54 (− 0.163, S.E. 0.039).

**Conclusion:**

There is reason to believe that the emergency policy response to the COVID-19 crisis may have had unintended and possibly scarring effects in terms of increased prevalence of insomnia. The hardest hit were young adults and, to a lesser extent, the middle-aged; however, older respondents (55 +) remained resilient, and their insomnia trajectory bounced back to pre-pandemic levels.

**Supplementary Information:**

The online version contains supplementary material available at 10.1186/s12889-022-14048-1.

## Background

According to the World Health Organization epidemiological update, as of early February 2022, about 400 million people have been diagnosed with the SARS-CoV-2 infection (COVID-19) and over 5.6 million people died from it throughout the world [1]. In addition to those who have been directly infected, many others have been affected by the psycho-social and economic consequences of COVID-19. From early in 2020, governments introduced measures to reduce the number of infections, including physical distancing, limits to freedom of movement both intra-and internationally and the closure of social spaces [2]. In addition to measuring the effects of these public health precautions against infection and mortality, which were central to society’s response to COVID-19, it is increasingly relevant to consider other costs associated with their widespread adoption [3]. Indeed, there is abundant evidence, produced internationally, of COVID-19-related psychological disorders (e.g. anxiety, depression and stress); reduced life satisfaction and domains satisfaction; and diminished happiness and flourishing [4–6]. Sleep has also been impaired [7–9].

There are a few mechanisms through which the pandemic could have affected sleep. Containment measures – such as lockdowns, quarantines, and mobility restrictions – have protected against the spread of the COVID-19 infection [10]; however, they have resulted in immediate and unintended detrimental effects. Workplace closures and changes in working times and rhythms dramatically altered daily schedules and routines, thus contributing to changes in sleep habits and patterns (in terms of wake-up times, sleep timing and sleep duration), which are associated with the circadian rhythms that regulate sleep [11–13]. Lack of regular exercise or, more generally, lower levels of physical activity following stay-at-home requirements reduced the need for sleep and led to difficulty falling asleep or to early wake-ups [14]. Physical distancing and confinement increased loneliness, raising perceived anxiety, and COVID-19 related worries, which in turn resulted in increased sleep disturbances [15–17]. Moreover, fear of illness has been widely documented in past research, especially the fear of contagion from infectious diseases [18–21]. During the COVID-19 pandemic, risk perception about own and others’ health and well-being increased, including the fear of falling ill or that a family member or friend would fall ill, the probability of contracting the COVID-19 or the fear of not surviving in the event of contracting COVID-19. As several studies have shown, the higher the level of perceived risk is, the worse the mental health and sleep disorders [22, 23]. In the scientific literature, the relationship between risk perception and poor sleep quality is well-documented, especially among healthcare workers [24, 25]. In the general population, Rossi and colleagues [26] highlighted a strong association between COVID-19 risk factors and severe insomnia symptoms in Italy. Blanc et al. [27] confirmed these results in their study analysing the relationship between risk perception and the Pittsburgh Sleep Quality Index (PSQI) in New York (US).

### Aim and objectives

Against this background, this study employed panel data on a sample of 883 individuals aged 16 + in Italy and investigated the COVID-19 related effects on insomnia, which is a condition of subjectively inadequate or non-restorative sleep [28]. First, we anticipated (Hypothesis 1) that the period of lockdown acted as a precipitating event and, like other stressful life experiences, could lead indirectly to the onset of insomnia [9, 13, 29]. Moreover, we intended to test the severity of sleep disturbances in the longer term. Therefore, we hypothesized (Hypothesis 2) that the gradual relaxation of containment measures, which took place in different policy phases, could possibly reduce insomnia and lead to a relative recovery towards baseline levels [30, 31]. Finally, we tested age-related heterogeneity in sleep responses to the health emergency and associated government restrictions. In greater detail, building on previous research which showed, even if not unequivocally [8, 32], that the severest psycho-social impact of the pandemic and policy measures to mitigate its impact have been produced on young people and adults [13, 33], we expected (Hypothesis 3) a significant increase in insomnia in the younger and middle-aged groups relative to their older counterparts.

## Methods

### Sample

**Fig. 1:**
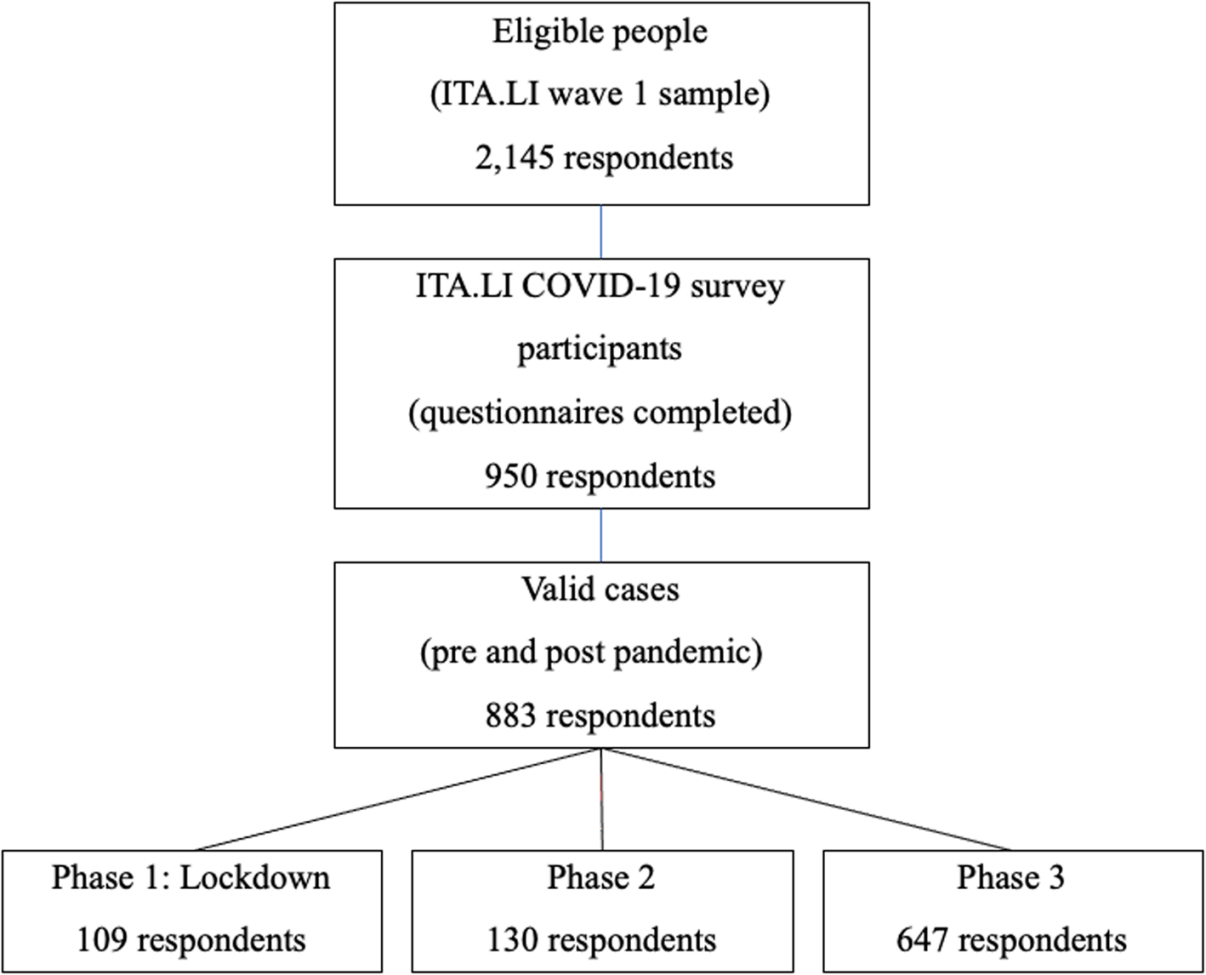
Flowchart of study design

Analyses drew on a subsample of respondents to ITA.LI – Italian Lives (hereafter ITA.LI), which is a recently established panel study based on a probability sample of 8,967 individuals aged 16 or older living in 4,900 households in Italy. From June 2019, it has collected crucial information on a wide set of topics, including family structures and dynamics, housing, education and employment, income and wealth and health and well-being. From April to September 2020, all panel members who had already taken part in ITA.LI wave 1 were invited to participate in a COVID-19 ad hoc survey (hereafter ITA.LI COVID-19). The ITA.LI COVID-19 survey, which was conducted using computer-aided web interviewing (CAWI) and computer-assisted telephone interviewing (CATI) methods [34], gathered information on the psychological, social and economic consequences of COVID-19 and mainly used items and measures already employed in ITA.LI wave 1. Of the 2,415 eligible people with direct contacts, 950 participated in the ITA.LI COVID-19 survey and completed the questionnaire. When merging data collected from ITA.LI wave 1 with the records of the respondents to ITA.LI COVID-19, data on 67 participants were dropped and excluded from the analysis because these were not matched to the previous wave of data collection or because of missing information on selected variables. Hence, 883 respondents were included in the regression analysis. The flowchart of the study design is shown in Fig. 1, and the sociodemographic characteristics of the sample are given in Table 1.

**Table 1:** Sample (*N* = 886)

Variable	Mean (SD)		Frequency (%)	
Age group				
16–34			144	(16.3)
35–54			342	(38.6)
55 or more			400	(45.1)
Gender				
Male			354	(40.0)
Female			532	(60.0)
Educational level				
Primary			280	(31.6)
Secondary			451	(50.9)
Tertiary			155	(17.5)
Cohabiting with partner or spouse				
Yes			507	(57.2)
No			379	(42.8)
Children living in the household by age group				
No children aged 0–14 years			731	(82.5)
Preschoolers			74	(8.4)
Children aged 7–14 years			81	(9.1)
Personality (Big Five)				
Extraversion	11.3	(1.9)		
Agreeableness	10.3	(2.1)		
Conscientiousness	11.7	(1.8)		
Neuroticism	9.1	(2.1)		
Openness	11.2	(2.1)		
Policy phase				
Phase1			109	(12.3)
Phase 2			130	(14.7)
Phase3			647	(73.0)

### Measures

*Insomnia* was assessed on a 3-point scale (0 “Not at all”; 1 “Somewhat”; 2 “Very much”), using the following question: “Have you experienced any difficulties in falling asleep or insomnia?”.

*Policy phases* were categorized as follows, based on the date of data collection in the ITA.LI COVID-19 survey and following key policy responses implemented at the national level: pre-pandemic (reference period), until 8 March 2020; first nation-wide lockdown (Phase 1), from 9 March until 3 May 2020, during which severe restrictions were in place to help stop the spread of COVID-19; Phase 2, from 4 May to 2 June 2020, in which several containment measures were relaxed, and retail shops, bar and restaurants and bars and personal services were reopened under COVID-19 safety guidelines; summer reopening (Phase 3), from 3 June 2020, onwards, in which most mandatory COVID-19 regulations, such as mobility restrictions across regions and restrictions to and from other EU countries, were lifted or removed.

Building on the relevant literature, gathered during COVID-19 and beyond [7, 29, 35], the following covariates were included in the models: *socio-demographic variables*: age (recoded in the following categories: 16–34; 35–54; 55 and older); gender (male; female); educational level (recoded in the following categories: primary, secondary, tertiary); cohabiting with a partner or spouse (no; yes); children living in the household by age group (recoded in the following categories: no children aged 0–14 years; pre-schoolers; children aged 7–14 years); and personality traits, measured with an Italian adaptation of the Big Five Inventory – Short version (BFI–S) [36]: agreeableness, extraversion, conscientiousness, neuroticism and openness.

### Statistical analysis

To estimate patterns of change in insomnia at different policy phases, we first fitted a random-effects ordered logistic model on the whole sample of respondents. This choice was justified by the ordered categorical nature of the outcome variable, which was measured at two time points for each respondent, one before the onset of the pandemic and one in the first six months (April–November 2020) of the pandemic. The model was specified as follows:

$${y}_{it}^{*}={\beta }^{\mathrm{^{\prime}}}{x}_{it}+{\alpha }_{i}+{\epsilon }_{it}\left(i=1,\dots , N;t=1,\dots T\right)$$

under the assumption:

$${\alpha }_{i}\sim N\left(0,{\sigma }^{2}\right); {\epsilon }_{it}\sim logistic\left(0,{\pi }^{2}/3\right);cov\left({\alpha }_{i, }{\epsilon }_{it}\right)$$

where ${y}_{it}^{*}$ denotes the latent continuous outcome of individual *i* on occasion *t*; a threshold model determines the observed response ${y}_{it}$ as follows:

$${y}_{it}=1 if {y}_{it}^{*}\le {k}_{1}; {y}_{it}=2 if {{k}_{1}<y}_{it}^{*}\le {k}_{2}; {y}_{it}=3 if { y}_{it}^{*}>{k}_{2};$$

to the ${k}_{th}$ category if ${\alpha }_{k-1}<{y}_{it}<{\alpha }_{k}$ where ${\alpha }_{k-1}$ and ${\alpha }_{k}$ denote the lower and upper boundaries of the ${k}_{th}$ category; ${x}_{it}$ is a set of observed covariates (and interaction terms) associated with the outcome; ${\alpha }_{i}$ is the individual-specific and time-invariant unobserved heterogeneity; and ${\epsilon }_{it}$ is the idiosyncratic error term.

We then added an interaction term between policy phases and the age of respondents to test whether the relationship between insomnia and the policy phases differed across individuals of different ages. Analyses accounted for ITA.LI COVID-19 survey nonresponse weights.

## Results

The analysis was based on 883 respondents out 2,415 people eligible for the ITA.LI COVID-19 survey (36.6%). A total of 109 respondents from ITA.LI wave 1 (12.3%) were re-interviewed during Phase 1; 130 (14.7%) during Phase 2; and finally, 647 (73.0%) in Phase 3. Of the respondents analysed, 6.3% were 16–34 years old, 38.6% aged 35–54 years and 45.1% 55 years or more; 60.0% were female, and 31.6% had a primary, 50.9% secondary and 17.5% tertiary education level. In terms of household composition, 57.2% were cohabiting and 82.5% had no children aged 0–14 years (Table 1). Summary statistics show that before the pandemic outbreak 64.3% of the total sample did not report any sleep disturbance. However, the same proportion reduced to 29.9% during Phase 1, i.e. confinement; it further reduced in Phase 2 (25.6%) and reached 51.9% in Phase 3. As for age groups, the proportion of individuals aged 16–34 reporting moderate or severe insomnia increased from 17.4% before the onset of COVID-19 to 58.3% in Phase 2 and to 64.5% in Phase 3, and it got to 41.4% in Phase 3. The prevalence of sleep disturbance among adults aged 35–54, which was 33.3% before the pandemic, augmented to 77.1% in Phase 1, 73.2% in Phase 2 and 47.5% in Phase 3. Finally, the fraction of adults aged 55 + suffering from moderate or severe insomnia raised from 44.4%, i.e. pre-pandemic levels, to 71.0% in Phase 1, to 85.3% in Phase 2, and it got to 50.3% in Phase 3.

**Fig. 2:**
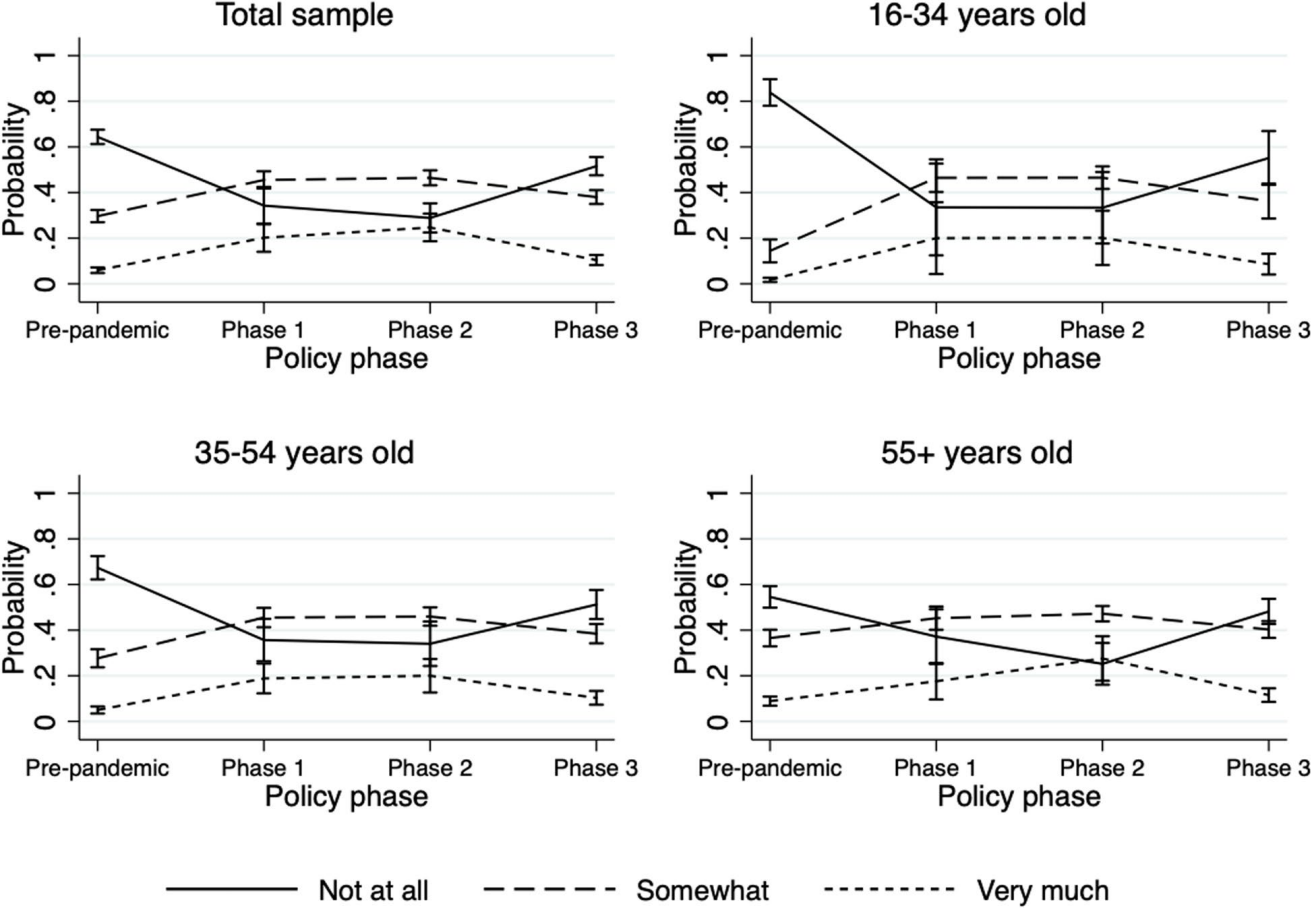
Predictive margins of policy phases on insomnia, by age groups

Table A1 in the Annex reports the parameter estimates, the estimated cut-off points and the estimated panel-level variance component of the random-effects ordered logistic models. To better and more easily interpret the results, we ran the “margins” command in STATA 17 and obtained the predicted probability for each of the three possible outcomes or categories of the outcome variable, for each level of the predictors (i.e. policy phase and the interaction between policy phase and age). We also computed the marginal change in the probability of each outcome. The predicted probabilities for the three categories of insomnia, which were plotted using the “marginsplot” command, are displayed in Fig. 2 (see also Table A2 in Annex for predicted probabilities and pairwise comparisons). Discrete changes in the probability of each outcome are reported in Table 2.

**Table 2:** Marginal effects of policy phases

	Marginal effect	SE	Unadjusted *p*-value	Unadjusted groups	Bonferroni *p*-value	Bonferroni groups
Insomnia: “not at all”						
Policy phase						
Phase 1	-0.301	0.042	0.000	-	-	-
Phase 2	-0.355	0.036	0.000	-	-	-
Phase 3	-0.128	0.024	0.000	-	-	-
Policy phase x Age group						
Phase 1 x 16–34	-0.486	0.103	0.000	A	0.000	A
Phase 1 x 35–54	-0.318	0.053	0.000	AB	0.000	AB
Phase 1 x 55 or more	-0.173	0.063	0.006	B	0.017	B
Phase 2 x 16–34	-0.487	0.088	0.000	A	0.000	A
Phase 2 x 35–54	-0.335	0.050	0.000	A	0.000	A
Phase 2 x 55 or more	-0.291	0.051	0.000	A	0.000	A
Phase 3 x 16–34	-0.269	0.060	0.000	A	0.000	A
Phase 3 x 35–54	-0.163	0.039	0.000	AB	0.000	AB
Phase 3 x 55 or more	-0.064	0.035	0.068	B	0.203	B
Insomnia: “somewhat”						
Policy phase						
Phase 1	0.159	0.017	0.000	-	-	-
Phase 2	0.168	0.015	0.000	-	-	-
Phase 3	0.084	0.016	0.000	-	-	-
Policy phase x Age group						
Phase 1 x 16–34	0.325	0.038	0.000		0.000	
Phase 1 x 35–54	0.176	0.025	0.000		0.000	
Phase 1 x 55 or more	0.084	0.024	0.000		0.001	
Phase 2 x 16–34	0.325	0.039	0.000		0.000	
Phase 2 x 35–54	0.180	0.023	0.000		0.000	
Phase 2 x 55 or more	0.100	0.023	0.000		0.000	
Phase 3 x 16–34	0.210	0.042	0.000		0.000	B
Phase 3 x 35–54	0.108	0.025	0.000		0.000	AB
Phase 3 x 55 or more	0.036	0.020	0.064		0.191	A
Insomnia: “very much”						
Policy phase						
Phase 1	0.142	0.030	0.000	-	-	-
Phase 2	0.187	0.030	0.000	-	-	-
Phase 3	0.045	0.010	0.000	-	-	-
Policy phase x Age group						
Phase 1 x 16–34	0.161	0.072	0.025	A	0.075	A
Phase 1 x 35–54	0.142	0.034	0.000	A	0.000	A
Phase 1 x 55 or more	0.090	0.041	0.030	A	0.091	A
Phase 2 x 16–34	0.162	0.056	0.004	A	0.011	A
Phase 2 x 35–54	0.155	0.037	0.000	A	0.000	A
Phase 2 x 55 or more	0.191	0.050	0.000	A	0.000	A
Phase 3 x 16–34	0.059	0.020	0.002	A	0.007	A
Phase 3 x 35–54	0.055	0.015	0.000	A	0.001	A
Phase 3 x 55 or more	0.027	0.016	0.079	A	0.238	A

This table reports discrete changes in respondents’ probability of experiencing no, moderate or severe insomnia at different policy phases (compared to the pre-pandemic baseline). Estimates sharing the same letter in the same phase-related group are not significantly different at the 5% level

On average, the fraction of respondents reporting moderate (“somewhat” + 0.159, S.E. 0.017) or severe (“very much” + 0.142, S.E. 0.030) sleep disturbance significantly increased during the lockdown period (Phase 1). Therefore Hypothesis 1 was supported. The gradual reopening following the lockdown period in Phase 2 did not bring about any significant immediate reduction in the proportion of sampled individuals having difficulties in falling asleep or staying asleep, which in fact further increased from pre-pandemic levels (“somewhat” + 0.168, S.E. 0.015; “very much” + 0.187, S.E. 0.030). However, as we can see from the curve illustrated in Fig. 2, in Phase 3 the prevalence of insomnia fell compared to that reported in previous policy phases, but it remained significantly higher than during the pre-pandemic period: the increase in the predicted probability of suffering from sleep loss was + 0.084 (S.E. 0.016) for the outcome “somewhat” and + 0.045 (S.E. 0.010) for the outcome “very much”. Thus Hypothesis 2 was partially supported.

Interaction tests indicated that changes in sleep patterns across policy phases were significantly different for respondents in different age groups. Before COVID-19, the predictive probability of an individual aged 16–34 not indicating any problem of insomnia at all was 0.860 (S.E. 0.027); however, it was 0.665 (S.E. 0.027) among respondents aged 35–54 and 0.537 (S.E. 0.025) among those 55 + . The probability of not suffering from insomnia significantly diminished for respondents in each age subgroup in the lockdown policy phase (Phase 1); accordingly, the reduction in the probability of this category was offset by an increase in the other two categories. That said, it is worth mentioning that sleep deterioration during the confinement (Phase 1) was significantly stronger for respondents aged 16–34, for whom the discrete change in the probability of not suffering at all from insomnia was − 0.486 (S.E. 0.103), than for their older counterparts, especially those aged 55 + (− 0.173, S.E. 0.063). In Phase 2, when containment measures were partially lifted, the overall prevalence of sleep disturbances remained substantially unchanged for each age subgroup, compared to that documented in the previous period, and pairwise comparisons showed that the predictive margins of reporting moderate or severe insomnia were not significantly different among the three age subgroups. Finally, during the summer reopening (Phase 3), the proportion of respondents indicating moderate or severe difficulty in initiating or maintaining sleep significantly fell for all age subgroups: the average predicted probability of not having reported sleep disturbances at all was 0.591 (S.E. 0.059) for respondents aged 16–34; 0.502 (S.E. 0.033) for those aged 35–54; and 0.473 (S.E. 0.029) for those aged 55 and over. Pairwise comparisons indicated that, because of these trends, the discrete change from pre-pandemic levels in the probability of not suffering from insomnia was negative and significant for the younger age group (− 0.269, S.E. 0.060) and for respondents aged 35–54 (− 0.163, S.E. 0.039); however, it was slightly negative but not statistically significant, at the 5% level, for the 55 + . Therefore, Hypothesis 3 was fully supported.

## Discussion

This study employed panel data techniques on data collected from a sample of respondents aged 16 + in Italy to investigate whether the COVID-19 pandemic resulted in sleep deterioration. Analyses were also focused on ascertaining if the gradual easing of containment measures was associated with significant improvement in sleep disturbances over a short time span (from April to September 2020) and on assessing possible age-related heterogeneity in pandemic-related effects on sleep.

The results confirmed previous research in the field and showed that, under COVID-19, average sleep quality declined compared to the pre-pandemic baseline, after adjusting for a set of common predisposing risk factors for loss of sleep. We could not test and demonstrate if the relatively higher prevalence of insomnia was a direct result of restrictions related to COVID-19 or if it was rather the consequence of increased worries, social isolation, fear of contagion or infection, stress or even the feeling of not being in control of the events that influence one’s own life, which has been found to negatively affect health and well-being [37]. Nonetheless, analyses fitted on individuals interviewed at different dates, which were indicative of different policy phases, showed that the quality of sleep followed a U-shaped trajectory over the course of the pandemic. In particular, the onset of COVID-19 and the implementation of lockdown (Phase 1) were associated with a sharp increase in the prevalence of moderate or severe insomnia, which did not recede in the so-called Phase 2, when containment measures were initially relaxed. Thus, the evidence collected points to the fact that a possible immediate improvement in the quality of life, resulting from the end of strict stay-at-home requirements, did not bring about an immediate recovery in the sample overall. A plausible explanation for this unexpected pattern is that even if people have the potential for resilience – that is, to report adaptive or better-than-expected outcomes despite significant risk and/or adversity [38, 39] – being able to improve and, particularly, to restore sleep requires time [40]. Recovery from sleep loss did not start until June 2020, but the pattern of recovery that emerged over the summer months (Phase 3) was only partial and ended up in a significant negative divergence from pre-pandemic levels. Hence, this study confirms previous research indicating that average sleep levels deteriorated within the first few months of the COVID-19 pandemic [7, 11]. It also contributes to the existing literature and shows that, despite steady improvements occurring over the summer of 2020, after the lifting of most COVID-19 containment measures, sleep did not bounce back to the pre-pandemic baseline. In other words, the negative effects that developed at the onset of COVID-19 apparently persisted over the course of the pandemic, at least within the short time span under investigation. It is worth noting that, due to the nature of the available data, we could not test whether this positive trajectory continued over the autumn and winter months of 2020 and eventually benefited from seasonal effects, despite an overall resurgence in COVID-19 infections and a second round of pandemic restrictions.

The impact of COVID-19 on insomnia is consistent with the results of several studies, although few of them highlighted age differences in sleep patterns and quality [40–42]. A considerable part of available research in the field has used cross-sectional and/or convenience samples [4], which prevents comparison of the change in the prevalence of sleep disturbances among people of different ages before and after the pandemic. The findings here thus revealed distinct patterns of change in sleep, based on the age of the respondents, over the first months of the pandemic. Those aged 16–34 experienced the sharpest increase in sleep disturbances at the very beginning during the period of national lockdown; adults aged 35–54 followed and reported the second-severest initial disruption of sleep compared to the pre-lockdown baseline. Sleep disturbances persisted and remained at the same levels in the subsequent policy phase. A subsequent recovery followed, which started later than expected and improved sleep. However, this pattern of bouncing back was experienced differently by the age subgroups: the prevalence of insomnia remained higher than the pre-pandemic baseline for younger and adult respondents, whereas it did not show any significant divergence from what was observed before the COVID-19 onset for the oldest adults.

Overall, respondents aged 16–34 were the worst affected, and such an acute deterioration in sleep added up to a marked decline in mental health that has already been recorded in previous research [43]. It must be emphasized that this group of young adults has been facing many normative transitions, such as changes in their educational and professional condition, peer and romantic relationships and other social and emotional experiences such as living alone for the first time, which are stressful [44]. The onset of the pandemic, along with the implementation of containment measures, and the consequent socio-economic outcomes may have complicated or interrupted these normative transitions, increasing stress and worsening sleep for young adults. For this subgroup, the only partial recovery of pre-lockdown sleep quality could be a hint that sleep disruption has long-term consequences. From a life course perspective, an exposure acting during a specific period could have lasting or lifelong effects on the structure or function of organs, tissues and body systems that are not modified in any dramatic way by later experiences. Adopting this “critical period” model [45], insomnia experienced during the pandemic could have a deeper effect on the health and wellbeing of young people over their lifetime than would be acknowledged in considering it as a transitory effect.

Despite significant findings, this study has a few limitations, in addition to those already discussed, which need to be acknowledged. First, we used a self-assessed measure of insomnia, which is therefore subject to recall bias and participants’ perceptions [46]. Second, we used a single-item, three-category self-report measure of sleep quality, which may have lacked sufficient variation and validity to accurately estimate effects, whereas existing questionnaires assessing the quality of sleep such as the Pittsburgh Sleep Quality Index (PSQI), the Athens Sleep Questionnaire (ASQ) or the National Sleep Foundation (NSF, USA) could overcome this limit. Third, we were not able to measure the duration of insomnia; however, disturbed sleep that lasts several nights to a month is considered short-term insomnia and is usually associated with a persistent stressful situation or environmental factors [28]. Fourth, there was a significant attrition rate between data collected in ITA.LI wave 1 and data collected in ITA.LI COVID-19. This means that follow-up data were not available for more than 60% of participants surveyed in wave 1 [47]. Fifth, although the longitudinal design and measurement of sleep disruption symptoms at different timepoints allows for prediction of changes in insomnia symptoms, the lack of manipulation of sleep limits causal interpretations.

## Conclusion

To the best of our knowledge, this is among the first studies to use panel data to investigate changes in the prevalence of insomnia under COVID-19 in Italy. Our analyses tracked changes in sleep disturbances before the pandemic outbreak, into the first lockdown period, and in the following six months when bans and restrictions were progressively lifted or removed. The results indicated increased levels of insomnia following the initial strict confinement that declined but persisted in the longer term. Hence, there is reason to believe that the emergency policy response to the COVID-19 crisis may have had scarring effects. In this respect, the hardest hit were the young adults (16–34), and, to a lower extent, adults aged 35–54; however, the older respondents (55 +) remained resilient, and their insomnia trajectory bounced back to pre-pandemic levels. Further research on the nature and extent of pandemic-related outcomes and on their association with mitigation measures is needed to inform and guide decision-makers, mitigate the unintended consequences of policy responses and prevent increasing social inequalities in a complex, highly uncertain and rapidly changing context.

## Supplementary Information

**Additional file 1:** **Table A1.** Random-effects ordered logistic models: parameter estimates, cut points and panel-level variance component. **Table A2.** Average predicted probabilities for insomnia-related issues at different policy phases and age groups.

## Data Availability

The datasets generated and analysed during the current study are not publicly available due to the necessity to protect data still under embargo. In accordance with EU General Data Protection Regulation (EU 2016/79) and the University of Milano-Bicocca internal regulations (D.R. 6256/2018, prot. 90,980/18), ITA.LI – Italian Lives data are fully encrypted, stored anonymously in the cloud and protected against unauthorized access, disclosure, modification or destruction. ITA.LI – Italian Lives data (specifically the ITA.LI – Italian Lives wave 1 and ITA.LI COVID-19 survey data used for this study) are currently available only to researchers working at the ITA.LI – Italian Lives project who have completed a registration process with the personal data protection officer, in accordance with the above-mentioned regulations. The same data will be made publicly available to researchers from the University of Milano-Bicocca and through Cross-National Equivalent File (https://www.cnefdata.org/) in due time. However, the data release policy (time of public release, details of how to apply for and access the datasets, end user licence, etc.) has not been formally defined yet. Due to this lack of formal policies and procedures, all data underlying the findings may be currently accessed, only for the purpose of reproducing the analyses, through the corresponding author (or any of the remaining authors) and the personal data protection officer at University of Milano-Bicocca. The personal data protection officer can be contacted (at rpd@unimib.it or certified email rpd@pec.unimib.it) for all queries concerning personal data processing and the exercise of any rights deriving from General Data Protection Regulation (EU 2016/79). The ITA.LI – Italian Lives Data Controller is the University of Milano-Bicocca, represented by its legal representative, the Rector Giovanna Iannantuoni (rettorato@unimib.it or certified email ateneo.bicocca@pec.unimib.it). All relevant materials that may reasonably be requested by others to reproduce the results will made available upon the publication of the study.
