# Supplementary material for: The prevalence of insomnia in different COVID-19 policy phases: Longitudinal evidence from ITA.LI – Italian Lives
Source: BMC Public Health. 2022 Sep 1;22:1657. doi: 10.1186/s12889-022-14048-1 (PMC9434520; doi:10.1186/s12889-022-14048-1)
Supplement: Supplementary file 1 — Additional file 1: Table A1. Random-effects ordered logistic models: parameter estimates, cut points and panel-level variance component. Table A2. Average predicted probabilities for insomnia-related issues at different policy phases and age groups. [file 12889_2022_14048_MOESM1_ESM.docx]

**Annex**

| **Table A1.** Random-effects ordered logistic models: parameter estimates, cut points and panel-level variance component | | | | |
| --- | --- | --- | --- | --- |
|  | Model 1 | | Model 2 | |
|  |  | |  | |
| Policy phase (ref.: pre-pandemic) |  |  |  |  |
| Phase 1 | 1.640*** | (0.248) | 3.036*** | (0.584) |
| Phase 2 | 1.966*** | (0.223) | 3.045*** | (0.567) |
| Phase 3 | 0.698*** | (0.134) | 1.856*** | (0.395) |
| Age group (ref.: 16-34) |  |  |  |  |
| 35-54 | 0.774*** | (0.227) | 1.436*** | (0.326) |
| 55 or more | 1.202*** | (0.232) | 2.150*** | (0.328) |
| Policy phase*Age group |  |  |  |  |
| Phase 1*35-54 |  |  | -1.277* | (0.650) |
| Phase 1*55 or more |  |  | -2.092** | (0.677) |
| Phase 2*35-54 |  |  | -1.191 | (0.628) |
| Phase 2*55 or more |  |  | -1.366* | (0.654) |
| Phase 3*35-54 |  |  | -0.954* | (0.444) |
| Phase 3*55 or more |  |  | -1.514*** | (0.434) |
| Gender (Ref.: Male) |  |  |  |  |
| Female | 0.605*** | (0.148) | 0.627*** | (0.153) |
| Educational level (ref.: Primary) |  |  |  |  |
| Secondary | -0.158 | (0.157) | -0.141 | (0.164) |
| Tertiary | -0.023 | (0.230) | -0.020 | (0.238) |
| Living with partner (ref.: Yes) |  |  |  |  |
| No | -0.400** | (0.148) | -0.419** | (0.153) |
| Living with children aged 0-14 (ref.: No) |  |  |  |  |
| 0-6 years | 0.401 | (0.316) | 0.432 | (0.329) |
| 7-14 years | -0.289 | (0.276) | -0.309 | (0.284) |
| Personality |  |  |  |  |
| Extraversion | -0.105* | (0.042) | -0.115** | (0.044) |
| Agreeableness | 0.056 | (0.040) | 0.063 | (0.042) |
| Conscientiousness | -0.007 | (0.043) | -0.006 | (0.045) |
| Neuroticism | 0.099** | (0.037) | 0.099* | (0.039) |
| Openness | 0.085* | (0.041) | 0.088* | (0.042) |
|  |  |  |  |  |
| Cut 1 | 2.834*** | (0.757) | 3.582*** | (0.812) |
| Cut 2 | 5.476*** | (0.776) | 6.293*** | (0.835) |
| σ_u_ | 1.257*** | (0.315) | 1.452*** | (0.347) |
| Robust standard errors in parentheses | |  |  |  |
| *** p<0.001, ** p<0.01, * p<0.05 | | | | |

Results are also controlled for sex, educational level, cohabiting with partner or spouse, children living in the household by age group, personality traits.

**Table A2.** Average predicted probabilities for insomnia-related issues at different policy phases and age groups

|  | predicted probabilities | SE | Unadjusted groups | | | | | | | | | | | | | | | | | |
| --- | --- | --- | --- | --- | --- | --- | --- | --- | --- | --- | --- | --- | --- | --- | --- | --- | --- | --- | --- | --- |
| Insomnia: “not at all” |  |  |  | | | | | | | | | | | | | | | | | |
| Pre-pandemic |  |  |  |  |  |  |  |  |  |  |  |  |  |  |  |  |  |  |  |  |
| 16-34 | 0.860 | 0.027 |  |  |  |  |  |  |  |  |  |  |  |  |  |  |  |  |  |  |
| 35-54 | 0.665 | 0.027 |  |  |  |  |  |  |  |  |  |  |  |  |  |  |  |  | Q |  |
| 55+ | 0.537 | 0.025 |  |  |  |  |  |  |  |  |  |  |  |  |  |  | O | P |  |  |
| Phase 1 |  |  |  |  |  |  |  |  |  |  |  |  |  |  |  |  |  |  |  |  |
| 16-34 | 0.374 | 0.112 |  |  |  |  | E | F | G | H | I | J | K | L | M | N | O | P |  |  |
| 35-54 | 0.346 | 0.047 |  |  |  |  |  | F |  |  | I | J | K | L | M | N |  |  |  |  |
| 55+ | 0.364 | 0.061 |  |  |  |  |  |  |  | H | I | J | K | L | M | N |  |  |  |  |
| Phase 2 |  |  |  |  |  |  |  |  |  |  |  |  |  |  |  |  |  |  |  |  |
| 16-34 | 0.372 | 0.083 |  |  |  |  | E |  | G |  | I | J | K | L | M | N | O |  |  |  |
| 35-54 | 0.330 | 0.049 |  |  |  |  |  |  | G |  | I | J | K | L |  |  |  |  |  |  |
| 55+ | 0.246 | 0.046 |  |  |  |  | E | F | G | H | I |  |  |  |  |  |  |  |  |  |
| Phase 3 |  |  |  |  |  |  |  |  |  |  |  |  |  |  |  |  |  |  |  |  |
| 16-34 | 0.591 | 0.059 |  |  |  |  |  |  |  |  |  |  |  |  |  |  |  | P | Q |  |
| 35-54 | 0.502 | 0.033 |  |  |  |  |  |  |  |  |  |  |  |  |  |  | O | P |  |  |
| 55+ | 0.473 | 0.029 |  |  |  |  |  |  |  |  |  |  |  |  |  | N | O | P |  |  |
| Insomnia “somewhat” |  |  |  |  |  |  |  |  |  |  |  |  |  |  |  |  |  |  |  |  |
| Pre-pandemic |  |  |  |  |  |  |  |  |  |  |  |  |  |  |  |  |  |  |  |  |
| 16-34 | 0.126 | 0.024 |  | B | C | D |  |  |  |  |  |  |  |  |  |  |  |  |  |  |
| 35-54 | 0.284 | 0.021 |  |  |  |  | E |  | G |  | I |  |  |  |  |  |  |  |  |  |
| 55+ | 0.371 | 0.019 |  |  |  |  |  |  |  |  |  | J | K | L | M |  |  |  |  |  |
| Phase 1 |  |  |  |  |  |  |  |  |  |  |  |  |  |  |  |  |  |  |  |  |
| 16-34 | 0.451 | 0.042 |  |  |  |  |  |  |  |  |  |  |  | L | M | N | O | P |  |  |
| 35-54 | 0.459 | 0.021 |  |  |  |  |  |  |  |  |  |  |  |  | M | N | O | P |  |  |
| 55+ | 0.454 | 0.025 |  |  |  |  |  |  |  |  |  |  |  |  | M | N | O | P |  |  |
| Phase 2 |  |  |  |  |  |  |  |  |  |  |  |  |  |  |  |  |  |  |  |  |
| 16-34 | 0.451 | 0.032 |  |  |  |  |  |  |  |  |  |  |  |  | M | N | O | P |  |  |
| 35-54 | 0.463 | 0.020 |  |  |  |  |  |  |  |  |  |  |  |  |  | N | O | P |  |  |
| 55+ | 0.471 | 0.018 |  |  |  |  |  |  |  |  |  |  |  |  |  | N | O | P |  |  |
| Phase 3 |  |  |  |  |  |  |  |  |  |  |  |  |  |  |  |  |  |  |  |  |
| 16-34 | 0.336 | 0.040 |  |  |  |  |  |  |  |  | I | J | K |  |  |  |  |  |  |  |
| 35-54 | 0.391 | 0.022 |  |  |  |  |  |  |  |  |  | J | K | L | M | N | O |  |  |  |
| 55+ | 0.407 | 0.019 |  |  |  |  |  |  |  |  |  |  | K | L | M | N | O |  |  |  |
| Insomnia: “very much” |  |  |  |  |  |  |  |  |  |  |  |  |  |  |  |  |  |  |  |  |
| Pre-pandemic |  |  |  |  |  |  |  |  |  |  |  |  |  |  |  |  |  |  |  |  |
| 16-34 | 0.014 | 0.004 |  |  |  |  |  |  |  |  |  |  |  |  |  |  |  |  |  |  |
| 35-54 | 0.052 | 0.008 | A |  |  |  |  |  |  |  |  |  |  |  |  |  |  |  |  |  |
| 55+ | 0.093 | 0.011 |  | B |  |  |  |  |  |  |  |  |  |  |  |  |  |  |  |  |
| Phase 1 |  |  |  |  |  |  |  |  |  |  |  |  |  |  |  |  |  |  |  |  |
| 16-34 | 0.175 | 0.073 | A | B | C | D | E | F | G |  |  |  |  |  |  |  |  |  |  |  |
| 35-54 | 0.194 | 0.034 |  |  |  | D | E | F |  |  |  |  |  |  |  |  |  |  |  |  |
| 55+ | 0.182 | 0.042 |  |  | C | D | E |  |  | H |  |  |  |  |  |  |  |  |  |  |
| Phase 2 |  |  |  |  |  |  |  |  |  |  |  |  |  |  |  |  |  |  |  |  |
| 16-34 | 0.176 | 0.056 |  | B | C | D | E |  |  |  |  |  |  |  |  |  |  |  |  |  |
| 35-54 | 0.207 | 0.038 |  |  |  | D | E | F | G |  |  |  |  |  |  |  |  |  |  |  |
| 55+ | 0.284 | 0.051 |  |  |  |  | E | F | G | H | I | J |  |  |  |  |  |  |  |  |
| Phase 3 |  |  |  |  |  |  |  |  |  |  |  |  |  |  |  |  |  |  |  |  |
| 16-34 | 0.073 | 0.020 | A | B |  |  |  |  |  |  |  |  |  |  |  |  |  |  |  |  |
| 35-54 | 0.107 | 0.016 |  | B | C |  |  |  |  |  |  |  |  |  |  |  |  |  |  |  |
| 55+ | 0.120 | 0.016 |  | B | C |  |  |  |  |  |  |  |  |  |  |  |  |  |  |  |
| Note: estimates sharing a letter in the group label are not significantly different at the 5% level | | | | | | | | | | | | | | | | | | | |  |
